# Mosquito- and tick-borne orthoflaviviruses cross an *in vitro* endothelial-astrocyte barrier

**DOI:** 10.3389/fcimb.2025.1624636

**Published:** 2025-07-02

**Authors:** Felix Schweitzer, Lara-Jasmin Schröder, Alina Friedrichs, Viktoria Gudi, Thomas Skripuletz, Imke Steffen, Martin Palus, Daniel Růžek, Albert Osterhaus, Chittappen Kandiyil Prajeeth

**Affiliations:** ^1^ Research Center for Emerging Infections and Zoonoses, University of Veterinary Medicine, Hannover, Germany; ^2^ Center for Systems Neuroscience (ZSN), Hannover, Germany; ^3^ Department of Neurology, Hannover Medical School, Hannover, Germany; ^4^ Institute of Biochemistry, University of Veterinary Medicine, Hannover, Germany; ^5^ Institute of Parasitology, Biology Centre of the Czech Academy of Sciences, Ceske Budejovice, Czechia; ^6^ Laboratory of Emerging Viral Infections, Veterinary Research Institute, Brno, Czechia; ^7^ Department of Experimental Biology, Faculty of Science, Masaryk University, Brno, Czechia

**Keywords:** orthoflavivirus, blood-brain barrier, endothelial cells, astrocytes, neuroinvasion, transendothelial electrical resistance

## Abstract

**Introduction:**

The genus *Orthoflavivirus* of the *Flaviviridae* family includes several notable pathogens such as mosquito-borne West-Nile virus (*Orthoflavivirus nilense*, WNV) and Tick-borne encephalitis virus (*Orthoflavivirus encephalitidis*, TBEV) that are highly neurotropic and may cause severe neurological disease leading to lifelong disabilities, coma and death. These viruses have developed mechanisms to breach the compact blood-brain barrier (BBB) and establish infection within the central nervous system (CNS). Nevertheless, neuroinvasive mechanisms of orthoflaviviruses remain poorly understood. Complex anatomy of the CNS and the organization of the BBB is a major challenge to study neuroinvasion of orthoflaviviruses *in vivo*. Therefore, *in vitro* BBB models are useful tools to study direct interaction of viruses with the endothelial barrier.

**Methods:**

In this study, we employed an *in vitro* transwell BBB model comprising primary mouse brain microvascular endothelial cells and astrocytes to compare the ability of mosquito-borne and tick-borne orthoflaviviruses to cross a compact endothelial barrier and reach the basolateral compartment of the transwell system. The influence of virus inoculation on the barrier properties was determined by measuring transendothelial electrical resistance (TEER).

**Results:**

The results demonstrate that while pathogenic WNV and TBEV cross the endothelial barrier the ability of low pathogenic Usutu virus (USUV) and Langat virus (LGTV) was inconsistent. All viruses tested display virus replication within the endothelial cells. Nevertheless, virus replication did not affect the barrier function of endothelial cells as demonstrated by sustained TEER and absence of leakage of high molecular weight dextran molecules through the endothelial barrier even at several hours post infection.

**Discussion:**

Our findings indicate that orthoflaviviruses can infect the endothelial cells, replicate within them without affecting the cells and its barrier function. Nevertheless, only pathogenic WNV and TBEV showed the ability to cross the endothelial barrier and reach the basolateral compartment.

## 1 Introduction

Changing climatic conditions and spread of vectors capable of transmitting disease have led to the emergence of several viral diseases in areas where such cases were previously not reported (Pradier et al., 2012; Mora et al., 2022; Ruscher et al., 2023). This includes viruses of the genus *Orthoflavivirus*, which consists of notable pathogens transmitted through arthropod vectors. Amongst these are mosquito-borne West-Nile virus (*Orthoflavivirus nilense*, WNV) and Tick-borne encephalitis virus (*Orthoflavivirus* encephalitidis, TBEV), known for their neurotropism and thus responsible for severe debilitating neurological disease (Mandl, 2005; Lim et al., 2011).

The genome of orthoflaviviruses is organized in a single open reading frame, which codes for one polyprotein and is further processed into three structural and seven non-structural proteins. The structural and non-structural proteins play pivotal role in the pathogenesis of orthoflaviviruses (Kuno et al., 1998; Neufeldt et al., 2018; Postler et al., 2023). Several orthoflaviviruses share a certain degree of amino acid sequence homology and exhibit varying levels of immune cross reactivity (Mandl et al., 1991; Iacono-Connors and Schmaljohn, 1992; Heinz and Stiasny, 2012; Rathore and St. John, 2020). Hence, they are grouped into distinct serogroups, which are also linked to their respective arthropod vectors. Within the mosquito-borne serogroup, the cluster of Japanese encephalitis virus (*Orthoflavivirus japonicum*, JEV) and its relatives, such as WNV, further includes Usutu virus (*Orthoflavivirus usutuense*, USUV). The latter causes disease in birds, but is virtually non-pathogenic in immunocompetent humans (Agliani et al., 2023). Similarly, in the tick-borne serogroup, Langat virus (*Orthoflavivirus langatense*, LGTV) is a close relative of TBEV sharing significant homology and is low- or non-pathogenic in humans. Hence, LGTV has been tested as live attenuated vaccine in the past. However, following vaccine trials conducted in early 1970’s using live LGTV, neurological complications were reported in few vaccinees. This highlighted the risk of using such attenuated strains as vaccines without proper understanding of the mechanism of viral neuropathogenesis (Smorodintsev and Dubov, 1986; Gritsun et al., 2003).

Studies have shown that after introduction to the vertebrate host, these viruses replicate at the peripheral sites and reach the central nervous system (CNS) following a viraemic phase (Ruzek et al., 2019; Habarugira et al., 2020). Since exchange across the compact blood-brain barrier (BBB) is highly regulated, invading pathogens or cellular infiltrates are usually excluded and hence the BBB forms an anatomical barrier to protect the CNS from adverse effects. While endothelial cells constitute the principal structural component of the BBB, astrocytes also are a critical part as they provide essential growth factors needed for maintaining barrier integrity. The compactness of the BBB is mainly attributed to tight-junction proteins, such as occludin, claudin-5 and *zonula occludens* protein (ZO-)1 that tightly bind the endothelial cells together. As a result pathogens are prevented from using paracellular spaces to gain access into the CNS (Kadry et al., 2020; Dunton et al., 2021). Nevertheless, several neurotropic viruses have evolved mechanisms to circumvent the compact BBB and enter the CNS. These include non-destructive mechanisms by which the viruses cross endothelial cells without causing any significant damage to the BBB. In this case, viruses use transcellular routes where they enter endothelial cells on the luminal side and are released into the CNS on the opposite side (Cain et al., 2019). On the other hand, viruses that have evolved more disruptive mechanisms do so either by modulating the expression of tight junction proteins as a consequence of inflammatory reaction or by inducing cell death by replicating within the endothelial cells. This leads to a loss of barrier integrity, thus allowing the viruses to take paracellular routes to reach the CNS (Wang et al., 2004; Rochfort et al., 2014). Based on the current evidence it remains speculative if initial neuroinvasion is non-disruptive, and the breakdown of the BBB during the course of infection is a consequence of subsequent neuroinflammation upon virus replication within the CNS.

In this study, we employed an *in vitro* transwell endothelial-astrocyte barrier model, to gain insights into the neuroinvasive mechanisms of mosquito- and tick-borne orthoflaviviruses such as WNV, USUV, TBEV and LGTV displaying varying degrees of pathogenicity for animals and humans.

## 2 Materials and methods

### 2.1 Ethics statement

Mice were maintained and bred in-house in compliance with European guidelines (EU directive on animal testing 2010/63/EU) and German Animal Welfare Law. The sacrifice of neonatal mice for collecting brain was performed after obtaining approvals from animal welfare authorities (approval no. TiHo-T-2023-12, TiHo-T-2024-14).

### 2.2 Viruses

WNV (strain NY99) was obtained from Martin Groschup, Friedrich-Loeffler-Institute, Greifswald, Germany and initial viral stocks were generated by propagating it in Vero cells. The source and propagation of TBEV (strain Neudoerfl) and LGTV (strain TP21) used in this study was previously described (Petry et al., 2021; Kubinski et al., 2023). USUV was isolated from a dead blackbird in Hannover, Germany and grown in PK-15 cells. Phylogenetic analysis showed that this isolate clustered along with Africa 2 strains of USUV (Störk et al., 2021). All viruses were propagated on BHK-21 cells by using an initial inoculum of 5×10^5^ TCID_50_/ml in T75 flasks. At three days post infection (dpi) culture supernatants were harvested, centrifuged at 1000xg for 10 minutes to remove cell debris and subsequently concentrated by centrifugation through *amicon^®^* Ultra-15 30k centrifugal filters (Merck Millipore, Darmstadt, Germany) at 2500xg for 15 minutes. Viral titers of the stocks generated were determined using the 50% tissue culture infectious dose (TCID_50_) on BHK-21 cells. Viral stocks were stored at -80°C until further use.

### 2.3 Primary mouse brain microvascular endothelial cells

C57BL/6 mouse primary brain microvascular endothelial cells (mBMEC) were obtained from *CellBiologics Inc.* (Chicago, IL, USA) and maintained in culture as per the manufacturer’s instructions. Initially, cells were grown in T25 culture flasks coated with gelatine-based coating solution (GCS, 0.1% ready-to-use solution; CellBiologics, Chicago, IL, USA) in complete mouse endothelial cell medium (CECM; CellBiologics, Chicago, IL, USA) until confluent. Following this, cells were harvested by trypsinization, transferred into T75 culture flasks and grown until confluent, and subsequently passaged in a similar manner. At the time of cell harvest, several aliquots of cells from each passage were frozen in CECM with additional fetal bovine serum (FBS, final 50%vol.; Thermo Fisher Scientific gibco, Waltham, MA, USA) and dimethyl sulfoxide (DMSO, final 10%vol.; Carl Roth, Karlsruhe, Germany) and stored at -150°C until further use. For the experiments endothelial cells were used between passage 3 to 5.

### 2.4 Primary astrocyte isolation

Primary mouse astrocytes were harvested from neonatal mouse brain mixed glia cultures as previously described (Prajeeth et al., 2018). Briefly, brains were collected from neonatal mice (P1-P4) after decapitation. Subsequently meninges were removed, and tissue was digested by enzymatic treatment with trypsin-EDTA (0.05%; Thermo Fisher Scientific gibco, Waltham, MA, USA) followed by treatment with bovine pancreas DNase (1mg/ml; Roche Merck, Darmstadt, Germany). Enzymatic reaction was stopped by application of soybean trypsin-inhibitor (10mg/ml; 50%vol.; Sigma-Aldrich Merck, Darmstadt, Germany)-based triturating solution (1%w/vol. bovine serum albumin (BSA; Sigma-Aldrich Merck, Darmstadt, Germany), 0.03%vol. DNase (Sigma-Aldrich Merck, Darmstadt, Germany) in PBS) and the suspension was filtered through a 70µm pore size sieve. Single cell suspensions obtained were seeded in poly-L-lysine (PLL, 100µg/ml; Sigma-Aldrich Merck, Darmstadt, Germany)-coated T75 flasks and cultured in Dulbecco’s Modified Eagle Medium (DMEM) supplemented with 4.5g/l D-glucose and L-glutamine (Thermo Fisher Scientific gibco, Waltham, MA, USA), FBS (final 10%vol.; Thermo Fisher Scientific gibco, Waltham, MA, USA) and 10,000 units of penicillin together with 10mg/ml streptomycin (PenStrep; Thermo Fisher Scientific gibco, Waltham, MA, USA) (MGP+ medium). After 14 to 21 days, flasks were treated with 10µM cytosine β-D-arabinofuranoside (ara-C; Thermo Fisher Scientific gibco, Waltham, MA, USA) for three days to eliminate proliferating microglia and oligodendrocyte precursor cells from the culture and the unaffected astrocytes cell layer was harvested by mild trypsinization (0.05% trypsin-EDTA for 10 min. at 37°C). At this point aliquots of cells were made in MGP+ medium with additional FBS (final 50%vol.; Thermo Fisher Scientific gibco, Waltham, MA, USA) and DMSO (final 10%vol.; Carl Roth, Karlsruhe, Germany) and stocks were stored at -150°C until further use in experiments. For each experiment, a fresh stock was thawed and cultured in a T75 flaks until confluency was reached, before cells were harvested as described above and adjusted cell solution was seeded onto transwell inserts as described below.

### 2.5 BHK-21 cells

Baby hamster kidney (BHK-)21 (C-13) cells (ATCC, CCL-10; Manassas, VA, USA) were cultured in minimal essential medium (including Earl’s salts and L-glutamine, Thermo Fisher Scientific gibco, Waltham, MA, USA) supplemented with PenStrep (1%vol.) and HEPES buffer solution (final concentration of 20mM; Thermo Fisher Scientific Gibco, Waltham, MA, USA) (MEM+H). For TCID_50_ assays, cells were harvested from cultures by trypsinization and seeded in 96-well plates at a density of 2×10^4^ cells per well, one day before assay.

### 2.6 *In vitro* transwell barrier model

As described by (Marshall et al., 2024). mBMECs and astrocytes were either cultured alone or in co-culture to establish a compact barrier model. For this purpose, polyester membranes of 24-well transwell inserts (pore size of 0.4µm; Corning^®^ costar^®^, Corning, NY, USA) were coated with GCS on the apical side and PLL on the basolateral side. For mBMECs-astrocytes co-cultures, 5×10^4^ astrocytes were seeded onto the PLL-coated basal side of the inserts and cultured for three days in MGP+ medium. At this point the apical side of the same inserts was coated with GCS and 5×10^4^ mBMECs were seeded in CECM onto the apical side. A day later inserts were transferred into a *cellZscope*-device (*cellZscope*+^®^, nanoAnalytics, Münster, Germany) and cultured until they were confluent. The *cellZscope* allowed real-time monitoring of transendothelial electrical resistance (TEER), values of which correspond to compactness and monolayer formation respectively. A plateau was observed in TEER at around 4 days in culture where the measured TEER was approximately 20-25Ωcm^2^ and at this point the cells formed a tight barrier. **Supplementary Figure 1** created using Biorender depicts the barrier model consisting of endothelial monoculture (**Supplementary Figure 1A**) and endothelial-astrocyte coculture on transwell inserts (**Supplementary Figure 1B**).

### 2.7 Barrier permeability assays

The compactness of the barrier model on transwell inserts was further assessed using a fluorescein isothiocyanate (FITC)-dextran barrier permeability assay as described in (Schweitzer et al., 2025). For this purpose, 70kDa FITC-dextran solution (Sigma Aldrich, St. Louis, MI, USA) was added at a final concentration of 1mg/ml to the apical side of inserts cultured with mBMEC in mono- or in co-culture with astrocytes after a desired TEER value (>20Ωcm^2^) was reached. Subsequently, culture supernatants from the basolateral compartments were collected at 0, 30, 60, 120 and 240 minutes after FITC-dextran application. For certain experiments the sampling of culture supernatants was done at 300 minutes after FITC-dextran application. Subsequently, fluorescence in the culture supernatants was measured (excitation wavelength: 485nm, emission wavelength: 535nm; Infinite^®^ 200 Pro plate reader, TECAN Group, Männedorf, Switzerland). To quantify translocated FITC-dextran a standard curve was generated by measuring fluorescence of a 2-fold dilution series of the FITC-dextran stock solution. Similarly, barrier permeability assays were also performed using inserts collected at 24 hours post inoculation (hpi) and 48hpi to assess the effect of virus inoculation.

### 2.8 Virus inoculation and translocation of barrier model

To the compact mBMEC-astrocyte co-cultures on transwell inserts 2.5×10^5^ TCID_50_ of WNV, TBEV, USUV, or LGTV were added respectively, to the apical compartment in 100µl of serum-free CECM. After one hour of inoculation at 37°C, the inoculum and basolateral supernatants were sampled and inserts were washed twice with CECM (apical side) and MGP+ medium (basolateral side), respectively, before fresh media were added to apical and basolateral compartments. Inserts were returned to the *cellZscope* and TEER was monitored over a period of up to 48hpi. At 24hpi and 48hpi, respectively, culture supernatants were collected from apical and basolateral compartments of respective inserts and viral titers within the culture supernatants were determined by TCID_50_ assays as described below. For determining the barrier integrity post infection, inserts were taken for the above-described barrier permeability assays.

### 2.9 Virus replication in endothelial cells

For assessing viral replication in brain endothelial cells, mBMECs (1×10^5^) were seeded into each well of 8-well chamber slides (SARSTEDT, Nümbrecht, Germany) coated with GCS. The following day, cells were infected with 5×10^5^ TCID_50_ of WNV, TBEV, USUV or LGTV respectively, for one hour at 37°C and 5% CO_2_ in serum-free CECM. After thoroughly washing the initial inoculum with serum-free CECM culture supernatants were collected from respective wells at regular intervals from 0hpi to 72hpi and at 144hpi. Viral titers in culture supernatants were determined using TCID_50_ assays. At each timepoint the respective chamber slide was washed thoroughly, fixed with paraformaldehyde (4% in PBS) for 20 minutes and used for immunocytochemistry analyses. Fixed cells were stored at 4°C in double-distilled water until immunocytochemistry assays were performed.

### 2.10 Virus quantification

Quantification of infectious viral particles of all sampled supernatants was done by TCID_50_ assays on BHK-21 cells and calculations were performed according to the method described by Reed and Muench (Reed and Muench, 1938). For this, supernatants were thawed and diluted in MEM+H (supplemented with 2% FBS instead of 10% FBS) as follows. Supernatants of the mBMEC monoculture infections in 8-well chamber slides were diluted 1:10, viral inoculums collected from the apical compartment of the inserts used in the transwell experiments were diluted 1:5, while post-infection apical compartment supernatants were diluted 1:2. Supernatants collected from the basolateral compartment at each time-point were not further diluted. Of these (diluted/undiluted) supernatants, 100µl per well were applied onto BHK-21 cells cultured in 96-well plates and serial dilutions were performed by transferring 10µl into the following well. Viral titers were quantified by microscopic read-out of the cytopathic effect (CPE) four days after supernatant application.

### 2.11 Data processing and statistical analysis

For comparison of insert-culture techniques, TEER values obtained at 72h and 120h after start of measurements were extracted and used for statistical analyses. A repeated measures (RM) one-way analysis of variance (ANOVA) with subsequent *Tukey’s test* for multiple comparison was performed on these values. For post-inoculation TEER presentation, TEER values obtained by automated *cellZscope* measurements of 2 inserts in 3 independent experimental replicates were synchronized by measurement timestamps after virus inoculation. Subsequently, TEER values obtained from virus-inoculated inserts were normalized to those obtained from mock-inoculated inserts by subtracting pre-inoculation differences, followed by normalization per timepoint to obtain relative TEER values. TEER values obtained at 24hpi and 48hpi were extracted and used for statistical analyses. FITC-dextran concentrations of the last taken measurements during barrier permeability assay (240 and 300 minutes after FITC-dextran application, respectively) were used for statistical analyses. Extracted TEER values and FITC-dextran concentrations obtained from virus-inoculated cultures were tested for significant difference to mock-inoculated inserts by performing a RM one-way ANOVA with subsequent *Dunnett’s test*. Viral titers determined in mBMEC mono-culture supernatants collected at 0hpi were considered as baseline viral titers and subsequently subtracted from respective viral titers determined in supernatants collected at the following timepoints. For statistical analyses of viral titers measured via TCID_50_ assay, obtained values were log_10_-transformed (Y=log_10_[Y]) and a RM two-way ANOVA was performed on the log_10_-transformed data. Subsequently, a *Tukey’s test* for multiple comparison was performed for each timepoint. For all statistical tests performed a p-value below 0.05 was considered statistically significant. All statistical analyses were performed using GraphPad Prism 10 software.

## 3 Results

### 3.1 *In vitro* barrier model

**Figure 1:**
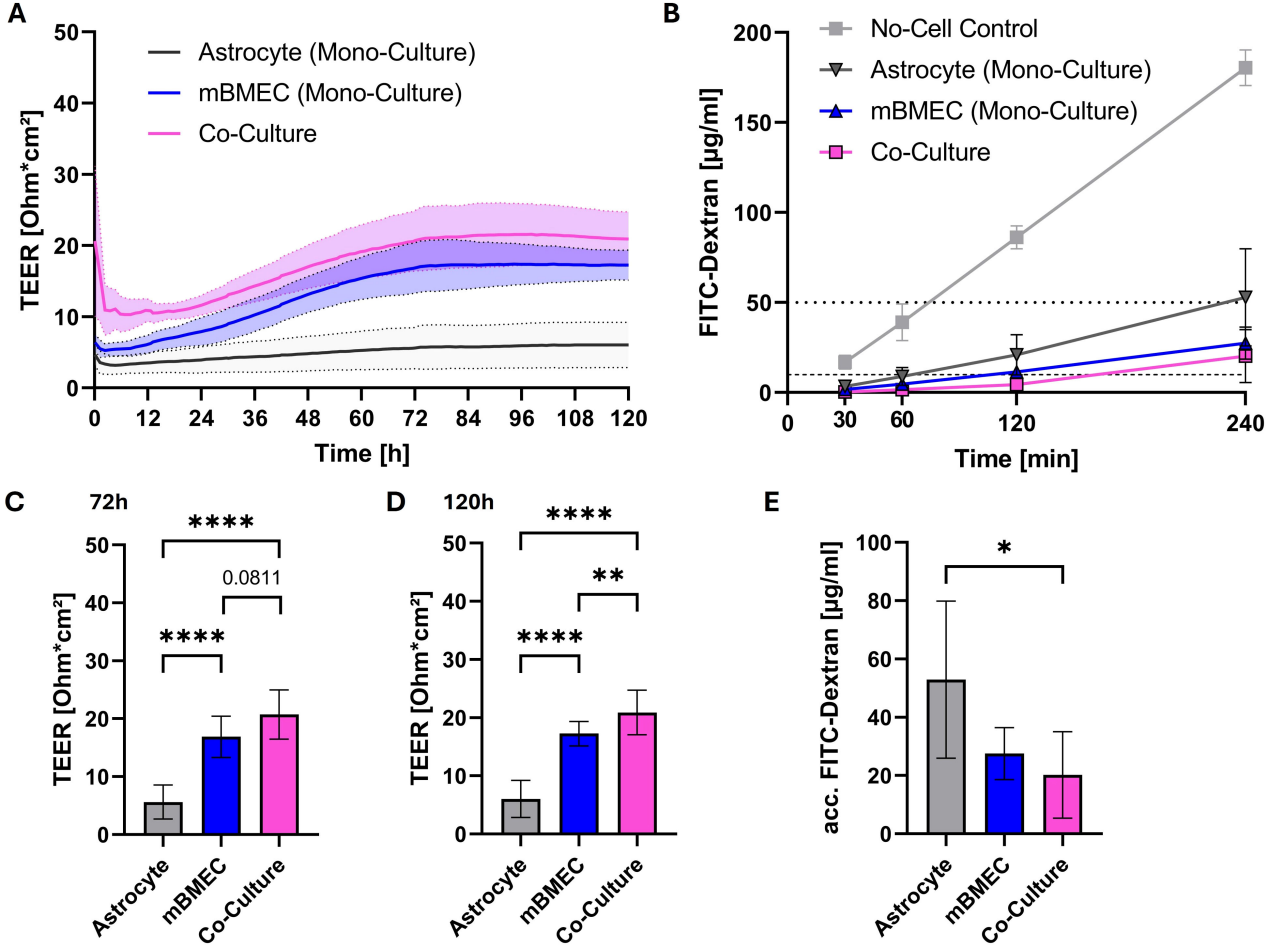
Transendothelial electrical resistance (TEER) monitored over time after introduction of transwell inserts to the *cellZscope* the day after seeding of mouse brain microvascular endothelial cells (mBMEC) and four days after astrocyte seeding. Presented are the mean values of n=6 inserts (continuous line) with the standard deviation (SD; dotted lines and filled area) **(A)**. TEER values obtained at 72h **(C)** and 120h **(D)** of incubation in the *cellZscope* were statistically tested with a repeated measures one-way analysis of variance (ANOVA), followed by *Tukey’s test* for multiple comparison **(C, D)**. Barrier permeability assay of inserts after 120h of incubation in the *cellZscope*. Presented are the values of n=3 inserts with the SD. Horizontal dotted line indicates 1% of FITC-dextran stock solution, whereas horizontal dashed line indicates 0.1% of applied FITC-dextran concentration **(B)**. FITC-dextran concentrations determined at 240 minutes after apical application were statistically tested with a repeated measures one-way ANOVA, followed by *Tukey’s test* for multiple comparison **(E)**. Asterisks indicate statistical significance as follows: p < 0.05*, p < 0.01**, p < 0.0001****.

To investigate the effects of orthoflaviviruses on barrier properties of brain endothelial cells *in vitro*, a transwell model was used in which compact monolayers of primary mouse brain microvascular endothelial cells (mBMEC) were cultured on the apical side of transwell inserts. To further enhance their barrier properties primary astrocytes were co-cultured on the basolateral side of the membrane. TEER measurements and barrier integrity assays were used to assess the compactness of the established barrier. TEER measurements showed significantly higher TEER in those inserts where mBMEC were co-cultured with astrocytes compared to those where only mBMEC were cultured on the transwell membrane. Monocultures with astrocytes alone did only show negligible electrical resistance (**Figure 1A**). Furthermore, co-cultures were less permeable to high molecular weight FITC-dextran (70 kda) compared to monocultures. The overall FITC-dextran concentration detected in the basolateral compartment of the co-cultures even after 240 minutes remained below 1% of the FITC-dextran concentration added to the apical compartment (**Figure 1**). Therefore, the mBMEC-astrocyte co-culture system was used for subsequent experiments.

### 3.2 Inoculation with orthoflaviviruses did not impact barrier properties

**Figure 2:**
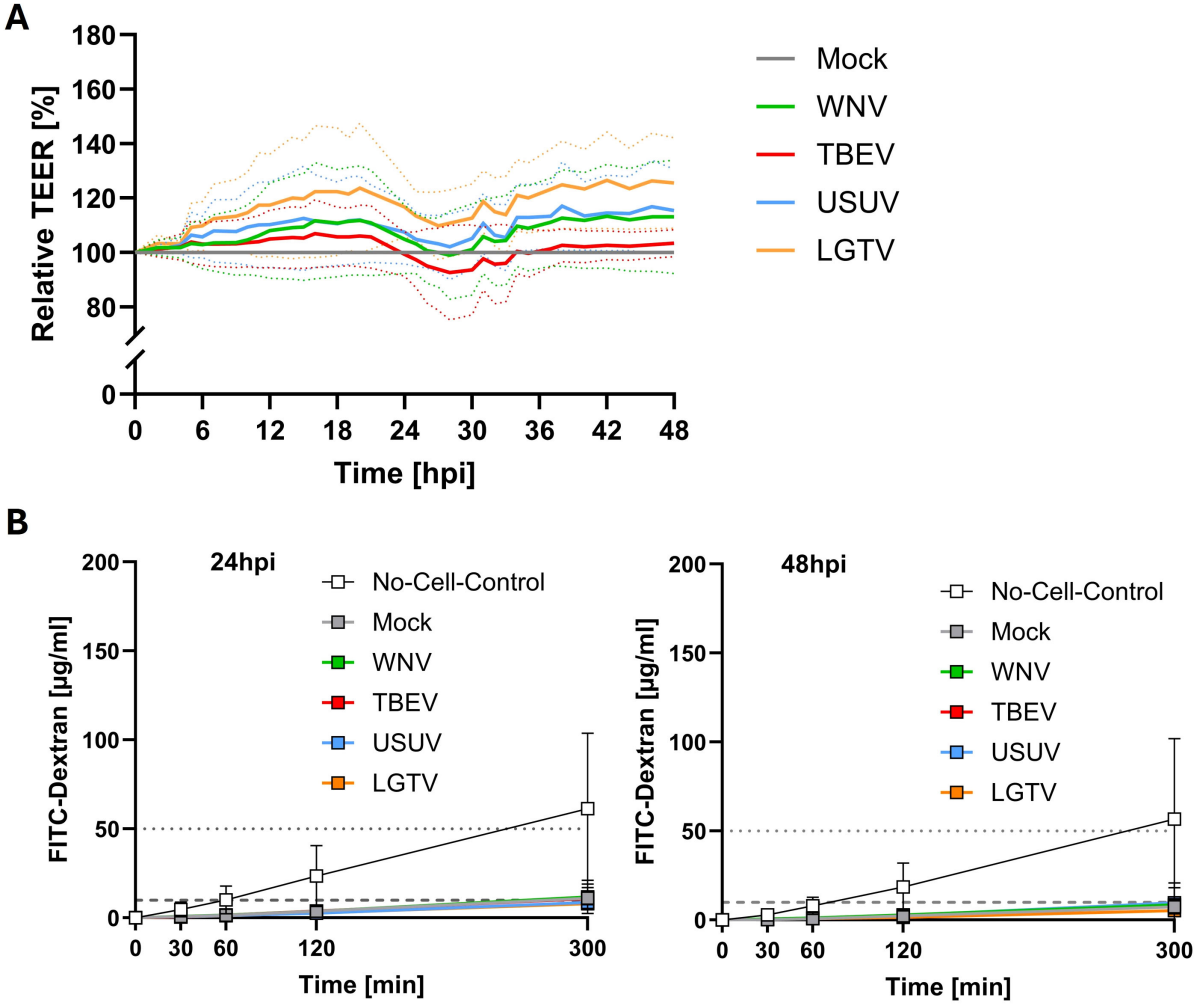
Relative transendothelial electrical resistance (TEER) monitored over time after inoculation of compact mBMEC-endothelial barriers on transwell inserts with West-Nile virus (WNV), Tick-borne encephalitis virus (TBEV), Usutu virus (USUV) and Langat virus (LGTV). Presented are the mean values of N=3 independent experiments (continuous line) with the standard deviation (SD; dotted lines) **(A)**. Barrier permeability assay of inserts at 24 and 48 **(B)** hours post inoculation with TBEV, LGTV, WNV and USUV. Presented are the mean values of N=3 independent experiments with the standard deviation (SD). Horizontal dotted line indicates 1% of FITC-dextran stock solution, whereas horizontal dashed line indicates 0.1% of applied FITC-dextran concentration **(B)**.

TEER values peaked around four days following culture of mBMEC on the apical side and astrocytes on the basolateral side of the transwell insert. At this point inserts were inoculated with WNV, TBEV, USUV, or LGTV on the apical side and the effect of the viruses on the barrier properties was assessed over a period of 48h post inoculation. Relative to mock inoculated inserts, a subtle increase in the TEER was observed initially in all inserts that were exposed to WNV, TBEV and USUV (**Figure 2A**). Only for LGTV inoculated inserts the increase in TEER was statistically significant compared to mock inoculated inserts (**Supplementary Figure 2**, p=0.0266 at 24hpi and p=0.0263 at 48hpi). Furthermore, the barrier integrity of the mBMEC-astrocyte barrier was maintained for all tested viruses as permeability of 70kDa FITC-dextran was highly restricted even at 24- and 48hpi (**Figure 2B**; **Supplementary Figure 3**). Application of staurosporine, an apoptosis-inducing agent, onto control inserts with a compact mBMEC-astrocyte barrier, in turn, led to substantial decrease of the TEER (**Supplementary Figure 4**). These findings suggest that the mBMEC-astrocyte barrier was intact and was not affected by infection with mosquito- (WNV & USUV) or tick-borne (TBEV & LGTV) orthoflaviviruses.

### 3.3 Translocation of mosquito- and tick-borne orthoflaviviruses across endothelial barrier follow different kinetics

**Figure 3:**
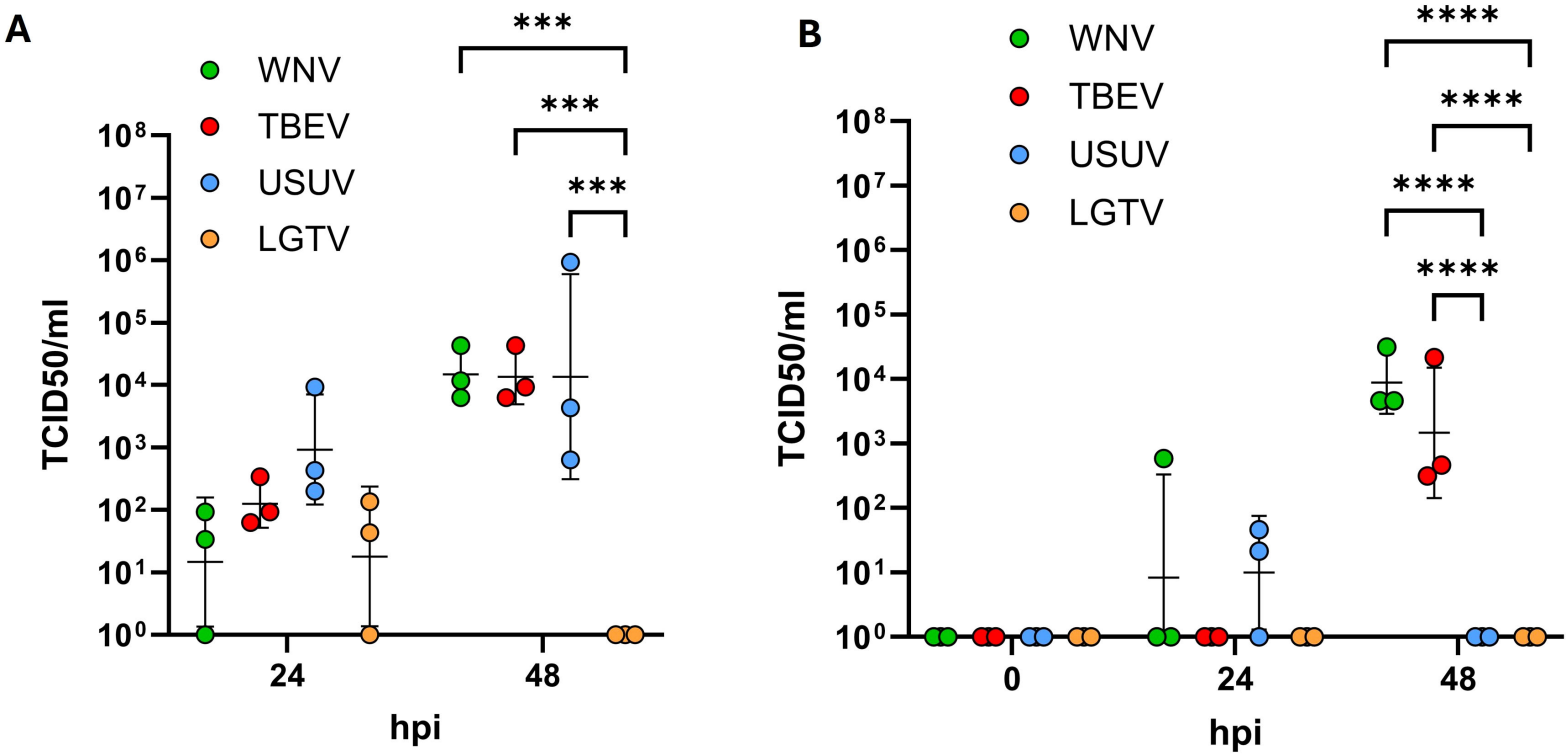
Quantification of viral particles in the apical **(A)** and basolateral **(B)** compartments via tissue culture infectious dose 50 (TCID_50_) assays. Assays were performed on supernatants collected at 24 and 48 hours post inoculation with West-Nile virus (WNV), Tick-borne encephalitis virus (TBEV), Usutu virus (USUV) and Langat virus (LGTV). Presented are the mean values of N=3 independent experiments with the standard deviation (SD). Original data was log-transformed and tested for statistical significance using a repeated measure two-way analysis of variance (ANOVA) followed by *Tukey’s test* for multiple comparison. Asterisks indicate statistical significance as follows: p < 0.001***, p < 0.0001****.

The findings so far indicate that orthoflaviviruses do not compromise the barrier integrity of the endothelial cells. We further investigated whether these viruses were prevented from crossing the endothelial barrier or employ non-disruptive transcellular routes to reach the basolateral compartments. For this purpose, one hour after virus inoculation on the apical side, inserts were thoroughly washed to remove residual input virus. Culture supernatants were then collected from both the apical and basolateral compartments at 24 and 48hpi and the viral titers were quantified using TCID_50_ assays. As shown in **Figure 3A**, at 24hpi all viruses were detected in the culture supernatants of the apical compartment indicating viral uptake and subsequent re-release into the same compartment. By 48hpi, increased titers of WNV, TBEV and USUV were observed in the apical compartment to be significantly higher than titers of LGTV (p=0.0002), which was not detected at this timepoint (**Figure 3A**). No viral particles were detected in the basolateral culture supernatants at 0hpi indicating that none of the viruses crossed the endothelial barrier during the inoculation period. At 24hpi no TBEV or LGTV was detected in the basolateral compartment whereas WNV was detected in one and USUV was detected in two out of three replicates. At 48hpi relatively high titers of WNV and TBEV were detected in the basolateral compartment and no USUV and LGTV were detectable at this timepoint (**Figure 3B**). Overall, compared to lower-pathogenic USUV and LGTV, WNV and TBEV showed the potential to cross the endothelial astrocyte barrier in significantly higher amounts (p<0.0001) nevertheless with delayed kinetics.

### 3.4 Orthoflaviviruses productively replicate within mBMEC mono-cultures

**Figure 4:**
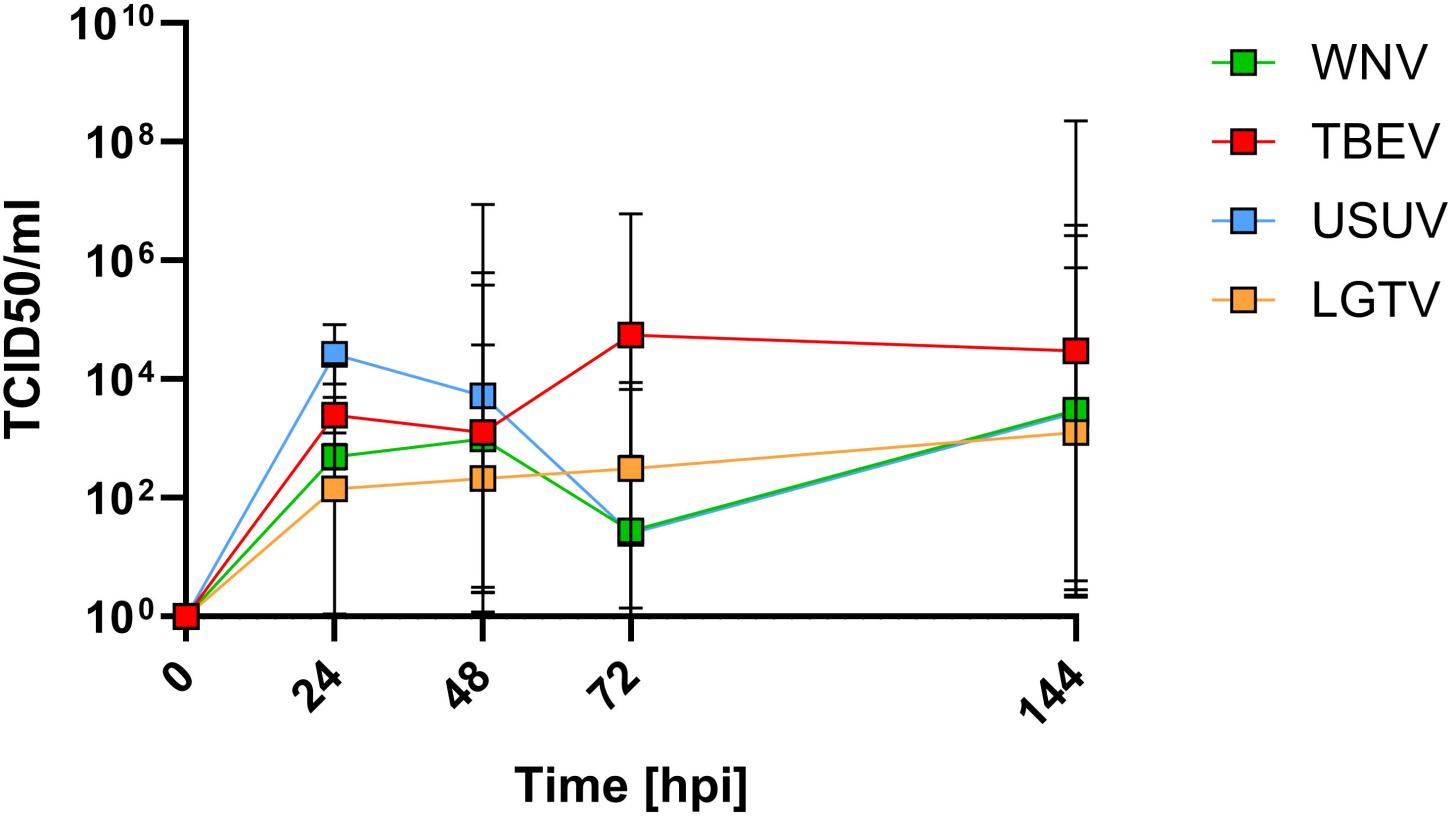
Quantification of infectious virus in the supernatants of mBMEC monocultures inoculated with TBEV, LGTV, WNV and USUV. TCID_50_ assays were performed on supernatants collected at different timepoints post inoculation. Presented are the mean values of N=3 independent experiments with the standard deviation (SD).

From the above results it is evident that WNV and TBEV demonstrated relatively superior ability to cross the endothelial-astrocyte barrier. To assess if this is due to different capacities of pathogenic versus less-pathogenic orthoflaviviruses to infect and replicate within the endothelial cells, mBMEC were inoculated with 510^5^ TCID_50_ WNV, TBEV, USUV and LGTV. Viral titers were determined from the culture supernatants collected at various time points post inoculation using TCID_50_ assays. The results suggest that all viruses replicate within the mBMEC albeit with different efficacies. Despite thorough washing to remove initial inoculum, small quantities of residual input virus were still detected in culture supernatants at 0hpi. Hence, for calculating the actual titers resulting from virus replication the background resulting from residual viruses in the culture supernatants was subtracted. As demonstrated in **Figure 4** for all tested viruses, increase in the viral titers was observed in the culture supernatants over the period of 144hpi indicating virus replication within mBMEC. Interestingly, TBEV showed slightly higher capacity to replicate within mBMEC compared to LGTV, WNV and USUV. Taken together these results indicate that both mosquito- and tick-borne orthoflaviviruses can infect and replicate within the endothelial cells, but also that the ability to cross the endothelial cell is not dependent on virus replication.

## 4 Discussion

In this study we compared the ability of neurotropic orthoflaviviruses with varying degrees of pathogenicity in humans and other mammalian species to cross a compact endothelial barrier and demonstrate that both mosquito- and tick-borne orthoflaviviruses cross a primary mouse endothelial cell barrier without compromising its integrity. The CNS is protected by compact and highly impermeable anatomical barriers such as the BBB, and as an initial step invading pathogens have to cross this barrier. Certain viruses such as ZIKV and Nipah virus (NiV) have been shown to infect and destroy the endothelial cells leading to vasculitis (Papa et al., 2017). Other viruses can cause BBB breakdown by triggering strong inflammation or endothelial cell death (German et al., 2006; Mishra et al., 2009; de Vries and Harding, 2023). In accordance with few previous reports we also demonstrate that WNV and TBEV cross the endothelial barrier and this occurs without compromising the endothelial barrier (Verma et al., 2009, 2010; Palus et al., 2017; Marshall et al., 2024). These findings are consistent with our recent findings which demonstrated that both TBEV and LGTV are capable of crossing human brain endothelial cell barrier without affecting its integrity (Schweitzer et al., 2025).

The BBB is a complex structure comprised of several other cell types such as astrocytes and pericytes in addition to tightly connected endothelial cells that essentially form a compact barrier. This implies that astrocyte and pericytes that are in close association with endothelial cells may be equally important in maintaining the integrity of the BBB (Pivoriūnas and Verkhratsky, 2021). In our preliminary analysis aimed at establishing a reliable barrier model, we have observed that transwell inserts with endothelial cell-astrocyte co-cultures provided relatively more compact barriers as demonstrated by improved TEER and decreased permeability compared to the inserts with endothelial cell mono-cultures. Several studies have shown that invading pathogens can modulate the properties of astrocytes and pericytes, which subsequently may contribute to the breakdown of the barrier. The best studied example among orthoflaviviruses is JEV which has been shown to infect both astrocytes and pericytes and induce proinflammatory factors such as IL-6 and matrix metalloproteases 2 and 9 which ultimately led to breakdown of endothelial barrier (Chen et al., 2014; Chang et al., 2015). Considering this, we employed an *in vitro* barrier model consisting of primary mouse brain endothelial cells and astrocytes to compare neuroinvasion of highly pathogenic WNV and TBEV and relatively non-pathogenic USUV and LGTV. For the viruses tested here, we did not see any notable changes in barrier properties despite of the presence of astrocytes. Similarly, in a recent study Marshall et al. used an *in vitro* BBB model consisting of human brain endothelial cells, astrocytes and pericytes to investigate neuroinvasion of WNV and USUV and demonstrated the ability of these viruses to cross the human brain endothelial cell barrier at different magnitudes without disrupting it (Marshall et al., 2024). TBEV strains Neudoerfl and Hypr with different pathogenic potentials were also shown to cross a human brain endothelial barrier model without altering its barrier properties (Palus et al., 2017). Interestingly, pathogenic viruses WNV and TBEV crossed the endothelial-astrocyte barrier despite an intact barrier whereas their non- or low-pathogenic counterparts for humans, USUV and LGTV respectively, were less efficient in overcoming the endothelial barrier and finding their way into basolateral compartment. The inability of 70 kDa FITC-dextran molecules to pass through endothelial barrier before and after infection supports the hypothesis that the viruses use transcellular routes to reach basolateral compartments rather than leaking through paracellular spaces. In this direction, using an *in vitro* human endothelial barrier model consisting of human cerebral microvascular endothelial cells (hCMEC/D3), we have demonstrated that TBEV and LGTV inoculated onto the apical side of the endothelial barrier transmigrate through hCMEC/D3 cells and are released on the basolateral side (Schweitzer et al., 2025). This process occurs without virus replication in the hCMEC/D3 cells. In contrast, in the current study we have observed that all viruses tested, WNV, TBEV, USUV and LGTV are capable of replicating within the mouse endothelial cells albeit with different efficacies. Though the ability to replicate within the endothelial cells was not significantly different between pathogenic and low- or non-pathogenic viruses the latter were inefficient in crossing the endothelial barrier. This implies that neuroinvasion most likely is not defined by the ability of viruses to replicate within the endothelial cells. Our observations are in line with the findings made by Marshall et al. where different efficiencies of WNV and USUV in crossing the endothelial cell barrier were attributed to their pathogenicity (Marshall et al., 2024). Immunofluorescence staining with antibodies directed against double stranded RNA (dsRNA) further supports the claim of efficient replication of pathogenic viruses within endothelial cells. We observed relatively higher dsRNA signal over time for TBEV and WNV, than the relatively low dsRNA signal for LGTV. Interestingly, dsRNA signals obtained for USUV are similar to those obtained for WNV (**Supplementary Figure 5**).

To our knowledge, this is the first study directly comparing the neuroinvasive properties of mosquito- and tick-borne orthoflaviviruses. Additionally, highly pathogenic viruses and closely related less pathogenic strains were used for comparison. Structurally both groups of orthoflaviviruses are similar, however, they cluster in distant genetic lineages (Neufeldt et al., 2018) and exhibit variations in viral proteins. For example, non-structural protein (NS)1 of mosquito-borne orthoflaviviruses such as DENV, JEV, WNV and ZIKV was shown to directly affect the integrity of human endothelial cell barriers *in vitro* (Puerta-Guardo et al., 2019). Although NS1 is conserved among orthoflaviviruses, its glycosylation sites differ between mosquito- and tick-borne orthoflaviviruses. These differences have been linked to complement interactions, endothelial glycocalyx layer disruption and neurovirulence (da Fonseca et al., 2017; Perera et al., 2023). Furthermore, NS1 of orthoflaviviruses is associated with vascular leakage (Puerta-Guardo et al., 2019). For this reason, we expected differences in the abilities of mosquito- and tick-borne orthoflaviruses to impact barrier properties. However, this proved not to be the case. A major limitation of *in vitro* endothelial barrier models is the low TEER values achieved, which does not accurately reflect the compactness of BBB observed *in vivo*. This is because BBB integrity *in vivo* is governed by several molecular and cellular factors and the extracellular matrix. Therefore, it is essential that such models are validated with appropriate methods, such as assessing the permeability of dextran molecules, to rule out the possibility of leakage through paracellular spaces. Nevertheless, well characterized and validated *in vitro* BBB models are useful tools to study direct effects of invading pathogens on the BBB.

Overall, the data obtained from this study suggests that initial neuroinvasive mechanisms employed by not only mosquito- but also tick-borne orthoflaviviruses are non-destructive and the viruses can enter the CNS without compromising the BBB. This has been demonstrated *in vivo* using a mouse model of TBEV infection, in which virus was detected in the brain before the BBB was compromised (Ruzek et al., 2011). Similarly, an increased permeability of the BBB is not necessary for a fatal outcome of WNV infection in mice (Morrey et al., 2008). In the absence of early BBB disruption during the initial neuroinvasion by pathogenic orthoflaviviruses, virus replication within target cells, innate immune sensing pathways and ensuing neuroinflammation are likely contributors to their neuropathogenesis. Finally, the endothelial cell-astrocyte co-culture model may provide an ideal platform to test and compare the neuroinvasive mechanisms of not only mosquito- and tick-borne orthoflaviviruses but also neurotropic viruses belonging to other families.

## Data Availability

The original contributions presented in the study are included in the article/**Supplementary Material**. Further inquiries can be directed to the corresponding author.

## Glossary

ANOVA: Analysis of variance
ara-C: Cytosine β-D-arabinofuranoside
BBB: Blood-brain barrier
BHK-21: Baby hamster kidney cells
BSA: Bovine serum albumin
CECM: Complete mouse endothelial cell medium
CNS: Central nervous system
CPE: Cytopathic effect
DMEM: Dulbecco’s Modified Eagle Medium
DMSO: Dimethyl sulfoxide
dsRNA: Double-stranded ribonucleic acid
FBS: Fetal bovine serum
FITC: Fluorescein isothiocyanate
GCS: Gelatin-based coating solution
hCMEC/D3: Human cerebral microvascular endothelial cells
hpi: Hours post inoculation
IL-6: Interleukin 6
JEV: Japanese encephalitis virus
LGTV: Langat virus
mBMEC: Mouse brain microvascular endothelial cells
MEM+H: Minimal essential medium supplemented with HEPES buffer
MGP+: Mixed glia culture preparation medium
MMP: Matrix metalloproteases
NiV: Nipah virus
NS1: Non-structural protein 1 (of orthoflaviviruses)
PLL: Poly-L-lysine
RM: Repeated measures
TBEV: Tick-borne encephalitis virus
TCID50: 50% Tissue culture infectious dose
TEER: Transendothelial electrical resistance
USUV: Usutu virus
WNV: West-Nile virus
ZIKV: Zika virus
ZO-1: Zonula occludens protein 1.

## References

[B1] Agliani G. Giglia G. Marshall E. M. Gröne A. Rockx B. H. G. van den Brand J. M. A. (2023). Pathological features of West Nile and Usutu virus natural infections in wild and domestic animals and in humans: A comparative review. One Health 16, 100525. doi: 10.1016/j.onehlt.2023.100525 37363223 PMC10288044

[B2] Cain M. D. Salimi H. Diamond M. S. Klein R. S. (2019). Mechanisms of pathogen invasion into the central nervous system. Neuron 103, 771–783. doi: 10.1016/j.neuron.2019.07.015 31487528

[B3] Chang C.-Y. Li J.-R. Chen W.-Y. Ou Y.-C. Lai C.-Y. Hu Y.-H.. (2015). Disruption of *in vitro* endothelial barrier integrity by Japanese encephalitis virus-Infected astrocytes. Glia 63, 1915–1932. doi: 10.1002/glia.22857 25959931

[B4] Chen C.-J. Ou Y.-C. Li J.-R. Chang C.-Y. Pan H.-C. Lai C.-Y.. (2014). Infection of pericytes *in vitro* by Japanese encephalitis virus disrupts the integrity of the endothelial barrier. J. Virol. 88, 1150–1161. doi: 10.1128/jvi.02738-13 24198423 PMC3911661

[B5] da Fonseca N. J. Lima Afonso M. Q. Pedersolli N. G. de Oliveira L. C. Andrade D. S. Bleicher L. (2017). Sequence, structure and function relationships in flaviviruses as assessed by evolutive aspects of its conserved non-structural protein domains. Biochem. Biophys. Res. Commun. 492, 565–571. doi: 10.1016/j.bbrc.2017.01.041 28087275

[B6] de Vries L. Harding A. T. (2023). Mechanisms of neuroinvasion and neuropathogenesis by pathologic flaviviruses. Viruses 15, 261. doi: 10.3390/v15020261 36851477 PMC9965671

[B7] Dunton A. D. Göpel T. Ho D. H. Burggren W. (2021). Form and function of the vertebrate and invertebrate blood-brain barriers. Int. J. Mol. Sci. 22, 12111. doi: 10.3390/ijms222212111 34829989 PMC8618301

[B8] German A. C. Myint K. S. A. Mai N. T. H. Pomeroy I. Phu N. H. Tzartos J.. (2006). A preliminary neuropathological study of Japanese encephalitis in humans and a mouse model. Trans. R. Soc. Trop. Med. Hygiene 100, 1135–1145. doi: 10.1016/j.trstmh.2006.02.008 16814333

[B9] Gritsun T. S. Lashkevich V. A. Gould E. A. (2003). Tick-borne encephalitis. Antiviral Res. 57, 129–146. doi: 10.1016/S0166-3542(02)00206-1 12615309

[B10] Habarugira G. Suen W. W. Hobson-Peters J. Hall R. A. Bielefeldt-Ohmann H. (2020). West nile virus: an update on pathobiology, epidemiology, diagnostics, control and “One health” Implications. Pathogens 9, 589. doi: 10.3390/pathogens9070589 32707644 PMC7400489

[B11] Heinz F. X. Stiasny K. (2012). Flaviviruses and their antigenic structure. J. Clin. Virol. 55, 289–295. doi: 10.1016/j.jcv.2012.08.024 22999801

[B12] Iacono-Connors L. C. Schmaljohn C. S. (1992). Cloning and sequence analysis of the genes encoding the nonstructural proteins of langat virus and comparative analysis with other flaviviruses. Virology 188, 875–880. doi: 10.1016/0042-6822(92)90545-Z 1316684

[B13] Kadry H. Noorani B. Cucullo L. (2020). A blood–brain barrier overview on structure, function, impairment, and biomarkers of integrity. Fluids Barriers CNS 17, 69. doi: 10.1186/s12987-020-00230-3 33208141 PMC7672931

[B14] Kubinski M. Beicht J. Zdora I. Saletti G. Kircher M. Petry-Gusmag M.. (2023). Cross-reactive antibodies against Langat virus protect mice from lethal tick-borne encephalitis virus infection. Front. Immunol. 14. doi: 10.3389/fimmu.2023.1134371 PMC10011100 36926332

[B15] Kuno G. Chang G.-J. J. Tsuchiya K. R. Karabatsos N. Cropp C. B. (1998). Phylogeny of the genus *flavivirus* . J. Virol. 72, 73–83. doi: 10.1128/JVI.72.1.73-83.1998 9420202 PMC109351

[B16] Lim S. M. Koraka P. Osterhaus A. D. M. E. Martina B. E. E. (2011). West nile virus: immunity and pathogenesis. Viruses 3, 811–828. doi: 10.3390/v3060811 21994755 PMC3185772

[B17] Mandl C. W. (2005). Steps of the tick-borne encephalitis virus replication cycle that affect neuropathogenesis. Virus Res. 111, 161–174. doi: 10.1016/j.virusres.2005.04.007 15871909

[B18] Mandl C. W. Iacono-Connors L. Wallner G. Holzmann H. Kunz C. Heinz F. X. (1991). Sequence of the genes encoding the structural proteins of the low-virulence tick-borne flaviviruses Langat TP21 and Yelantsev. Virology 185, 891–895. doi: 10.1016/0042-6822(91)90567-U 1720591

[B19] Marshall E. M. Koopmans M. Rockx B. (2024). Usutu virus and West Nile virus use a transcellular route of neuroinvasion across an *in vitro* model of the human blood–brain barrier. NPJ Viruses 2, 1–9. doi: 10.1038/s44298-024-00034-4 40295794 PMC11721115

[B20] Mishra M. K. Dutta K. Saheb S. K. Basu A. (2009). Understanding the molecular mechanism of blood–brain barrier damage in an experimental model of Japanese encephalitis: Correlation with minocycline administration as a therapeutic agent. Neurochemistry Int. 55, 717–723. doi: 10.1016/j.neuint.2009.07.006 19628016

[B21] Mora C. McKenzie T. Gaw I. M. Dean J. M. von Hammerstein H. Knudson T. A.. (2022). Over half of known human pathogenic diseases can be aggravated by climate change. Nat. Clim. Change 12, 869–875. doi: 10.1038/s41558-022-01426-1 PMC9362357 35968032

[B22] Morrey J. D. Olsen A. L. Siddharthan V. Motter N. E. Wang H. Taro B. S.. (2008). Increased blood–brain barrier permeability is not a primary determinant for lethality of West Nile virus infection in rodents. J. Gen. Virol. 89, 467–473. doi: 10.1099/vir.0.83345-0 18198377

[B23] Neufeldt C. J. Cortese M. Acosta E. G. Bartenschlager R. (2018). Rewiring cellular networks by members of the Flaviviridae family. Nat. Rev. Microbiol. 16, 125–142. doi: 10.1038/nrmicro.2017.170 29430005 PMC7097628

[B24] Palus M. Vancova M. Sirmarova J. Elsterova J. Perner J. Ruzek D. (2017). Tick-borne encephalitis virus infects human brain microvascular endothelial cells without compromising blood-brain barrier integrity. Virology 507, 110–122. doi: 10.1016/j.virol.2017.04.012 28432926

[B25] Papa M. P. Meuren L. M. Coelho S. V. A. Lucas C. G. de O. Mustafá Y. M.. (2017). Zika virus infects, activates, and crosses brain microvascular endothelial cells, without barrier disruption. Front. Microbiol. 8. doi: 10.3389/fmicb.2017.02557 PMC5743735 29312238

[B26] Perera D. R. Ranadeva N. D. Sirisena K. Wijesinghe K. J. (2023). Roles of NS1 protein in flavivirus pathogenesis. ACS Infect. Diseases. 10 (1), 20–56. doi: 10.1021/acsinfecdis.3c00566 38110348

[B27] Petry M. Palus M. Leitzen E. Mitterreiter J. G. Huang B. Kröger A.. (2021). Immunity to TBEV Related Flaviviruses with Reduced Pathogenicity Protects Mice from Disease but Not from TBEV Entry into the CNS. Vaccines 9, 196. doi: 10.3390/vaccines9030196 33652698 PMC7996866

[B28] Pivoriūnas A. Verkhratsky A. (2021). Astrocyte–endotheliocyte axis in the regulation of the blood–brain barrier. Neurochem. Res. 46, 2538–2550. doi: 10.1007/s11064-021-03338-6 33961207

[B29] Postler T. S. Beer M. Blitvich B. J. Bukh J. de Lamballerie X. Drexler J. F.. (2023). Renaming of the genus Flavivirus to Orthoflavivirus and extension of binomial species names within the family Flaviviridae. Arch. Virol. 168, 224. doi: 10.1007/s00705-023-05835-1 37561168

[B30] Pradier S. Lecollinet S. Leblond A. (2012). West Nile virus epidemiology and factors triggering change in its distribution in Europe. Rev. Sci. Tech 31, 829–844. doi: 10.20506/rst.31.3.2167 23520737

[B31] Prajeeth C. K. Dittrich-Breiholz O. Talbot S. R. Robert P. A. Huehn J. Stangel M. (2018). IFN-γ Producing th1 cells induce different transcriptional profiles in microglia and astrocytes. Front. Cell. Neurosci. 12. doi: 10.3389/fncel.2018.00352 PMC6191492 30364000

[B32] Puerta-Guardo H. Glasner D. R. Espinosa D. A. Biering S. B. Patana M. Ratnasiri K.. (2019). Flavivirus NS1 triggers tissue-specific vascular endothelial dysfunction reflecting disease tropism. Cell Rep. 26, 1598–1613.e8. doi: 10.1016/j.celrep.2019.01.036 30726741 PMC6934102

[B33] Rathore A. P. S. St. John A. L. (2020). Cross-reactive immunity among flaviviruses. Front. Immunol. 11. doi: 10.3389/fimmu.2020.00334 PMC7054434 32174923

[B34] Reed L. J. Muench H. (1938). A simple method of estimating fifty per cent endpoints. Am. J. Epidemiol. 27, 493–497. doi: 10.1093/oxfordjournals.aje.a118408

[B35] Rochfort K. D. Collins L. E. Murphy R. P. Cummins P. M. (2014). Downregulation of blood-brain barrier phenotype by proinflammatory cytokines involves NADPH oxidase-dependent ROS generation: consequences for interendothelial adherens and tight junctions. PloS One 9, e101815. doi: 10.1371/journal.pone.0101815 24992685 PMC4081725

[B36] Ruscher C. Patzina-Mehling C. Melchert J. Graff S. L. McFarland S. E. Hieke C.. (2023). Ecological and clinical evidence of the establishment of West Nile virus in a large urban area in Europe, Berlin, Germany 2021 to 2022. Eurosurveillance 28, 2300258. doi: 10.2807/1560-7917.ES.2023.28.48.2300258 38037727 PMC10690859

[B37] Ruzek D. Avšič Županc T. Borde J. Chrdle A. Eyer L. Karganova G.. (2019). Tick-borne encephalitis in Europe and Russia: Review of pathogenesis, clinical features, therapy, and vaccines. Antiviral Res. 164, 23–51. doi: 10.1016/j.antiviral.2019.01.014 30710567

[B38] Ruzek D. Salát J. Singh S. K. Kopecký J. (2011). Breakdown of the blood-brain barrier during tick-borne encephalitis in mice is not dependent on CD8+ T-cells. PloS One 6, e20472. doi: 10.1371/journal.pone.0020472 21629771 PMC3100324

[B39] Schweitzer F. Letoha T. Osterhaus A. Prajeeth C. K. (2025). Impact of tick-borne orthoflaviviruses infection on compact human brain endothelial barrier. Int. J. Mol. Sci. 26, 2342. doi: 10.3390/ijms26052342 40076965 PMC11901142

[B40] Smorodintsev A. A. Dubov A. V. (1986). The correlation between the pathogenesis and biological characteristics of tick-borne encephalitis virus. Tick-borne encephalitis its vaccino-prophylaxis Medicine Moscow 113–124, 190–211.

[B41] Störk T. de le Roi M. Haverkamp A.-K. Jesse S. T. Peters M. Fast C.. (2021). Analysis of avian Usutu virus infections in Germany from 2011 to 2018 with focus on dsRNA detection to demonstrate viral infections. Sci. Rep. 11, 24191. doi: 10.1038/s41598-021-03638-5 34921222 PMC8683490

[B42] Verma S. Kumar M. Gurjav U. Lum S. Nerurkar V. R. (2010). Reversal of West Nile virus-induced blood–brain barrier disruption and tight junction proteins degradation by matrix metalloproteinases inhibitor. Virology 397, 130–138. doi: 10.1016/j.virol.2009.10.036 19922973 PMC3102050

[B43] Verma S. Lo Y. Chapagain M. Lum S. Kumar M. Gurjav U.. (2009). West Nile virus infection modulates human brain microvascular endothelial cells tight junction proteins and cell adhesion molecules: Transmigration across the *in vitro* blood-brain barrier. Virology 385, 425–433. doi: 10.1016/j.virol.2008.11.047 19135695 PMC2684466

[B44] Wang T. Town T. Alexopoulou L. Anderson J. F. Fikrig E. Flavell R. A. (2004). Toll-like receptor 3 mediates West Nile virus entry into the brain causing lethal encephalitis. Nat. Med. 10, 1366–1373. doi: 10.1038/nm1140 15558055

