# Supplementary figures and images for: Mosquito- and tick-borne orthoflaviviruses cross an in vitro endothelial-astrocyte barrier
Source: Front Cell Infect Microbiol. 2025 Jul 2;15:1624636. doi: 10.3389/fcimb.2025.1624636 (PMC12263646; doi:10.3389/fcimb.2025.1624636)

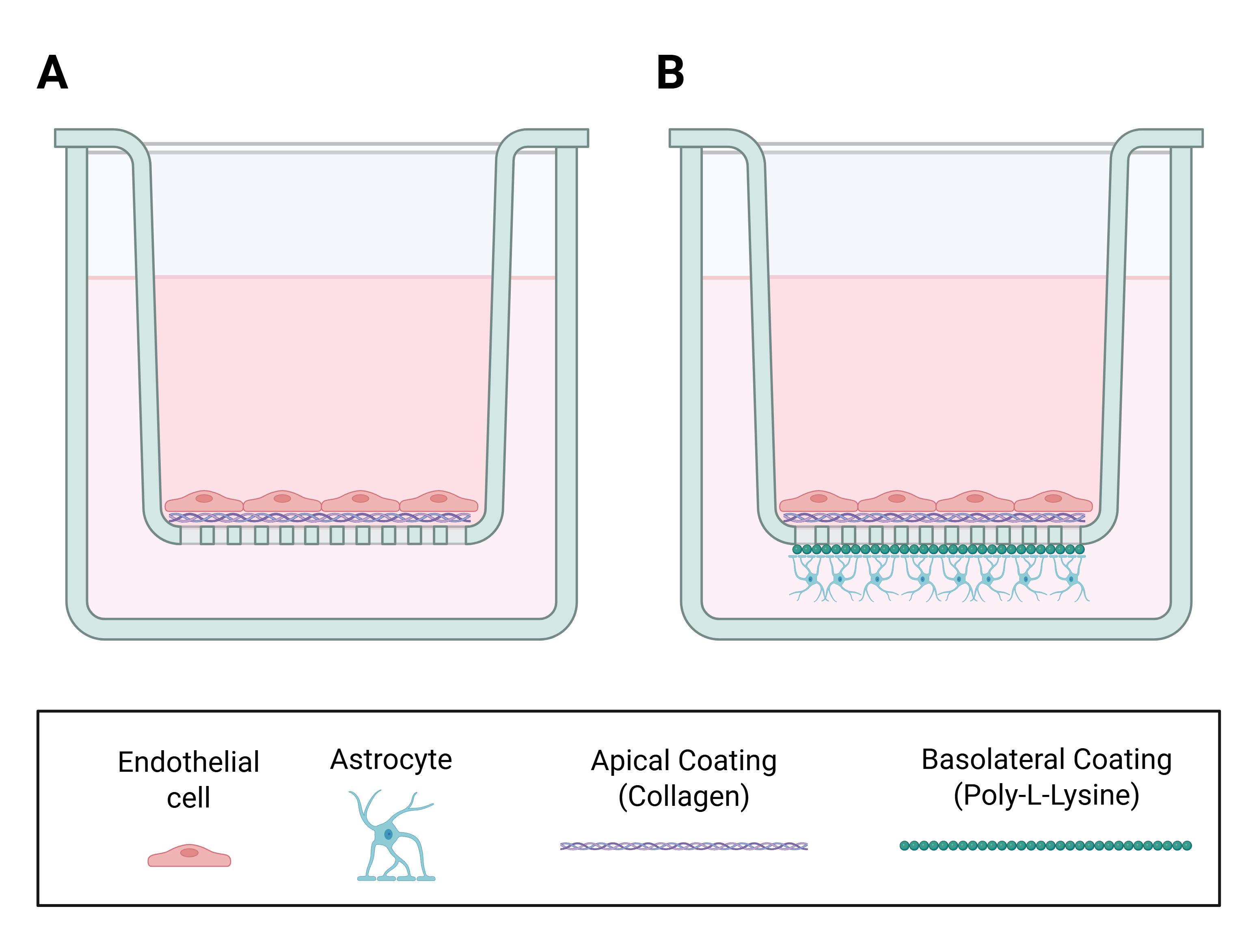

Supplement: Supplementary file 1 [file Image1.tif]

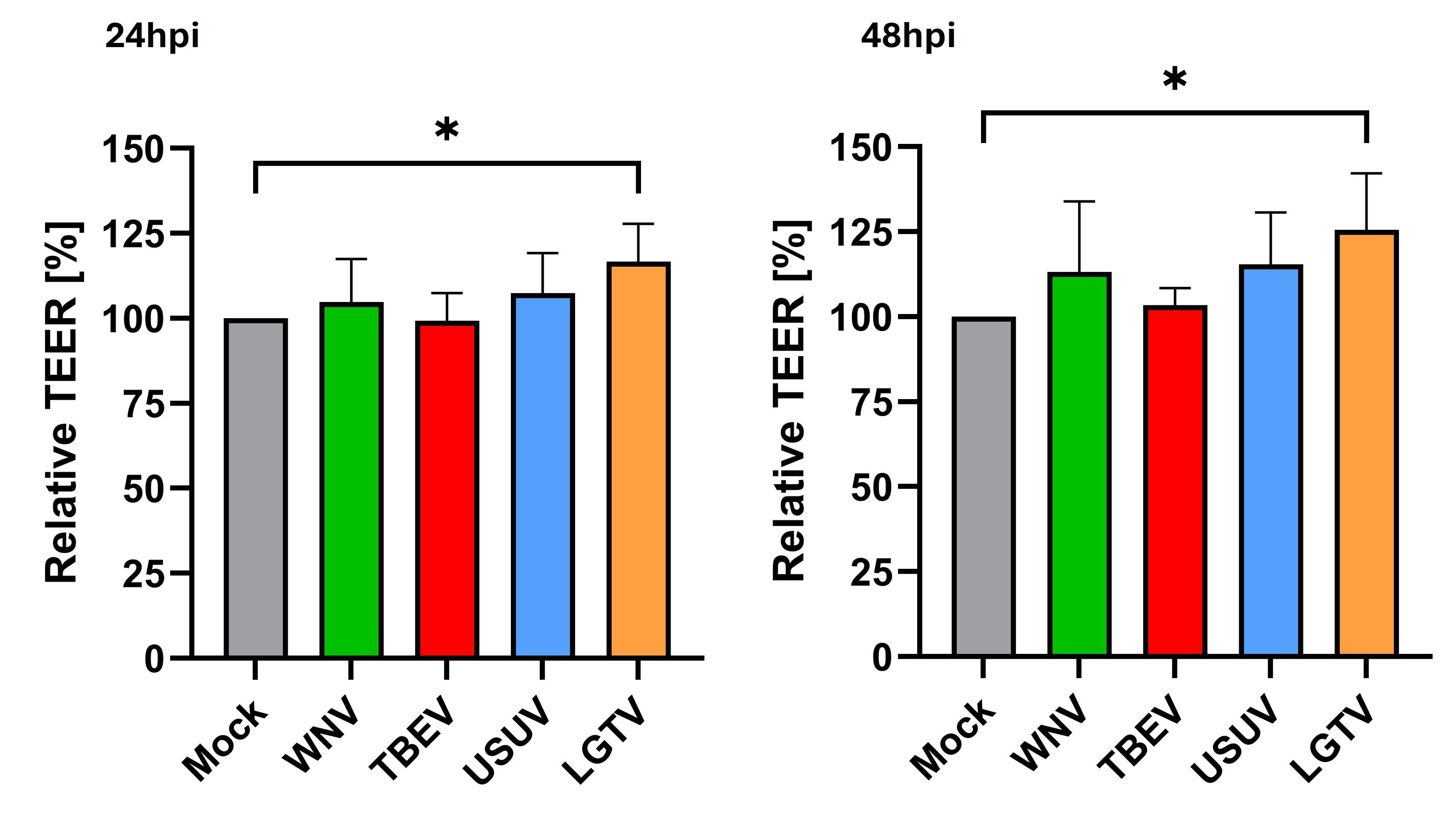

Supplement: Supplementary file 2 [file Image2.tif]

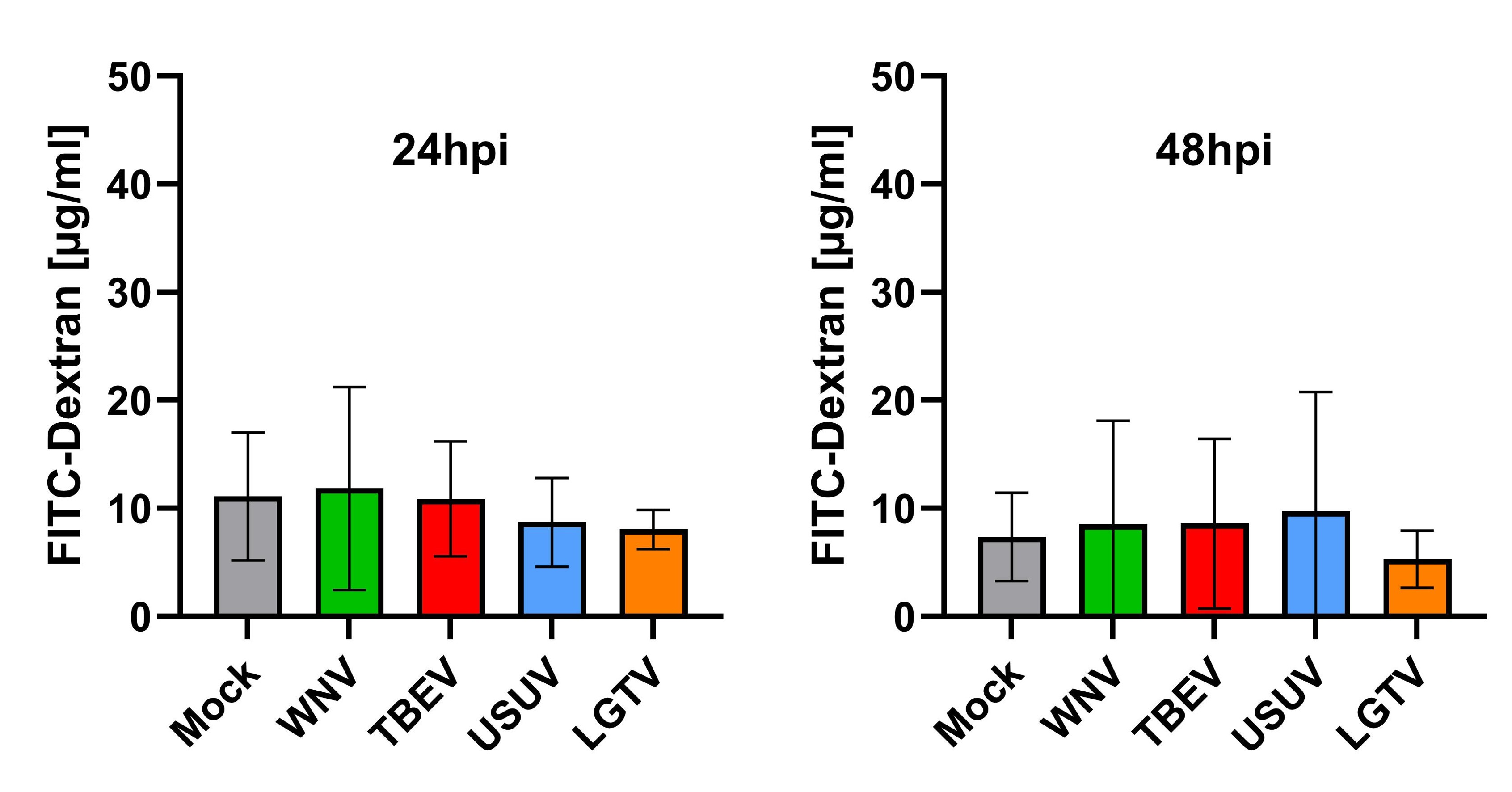

Supplement: Supplementary file 3 [file Image3.tif]

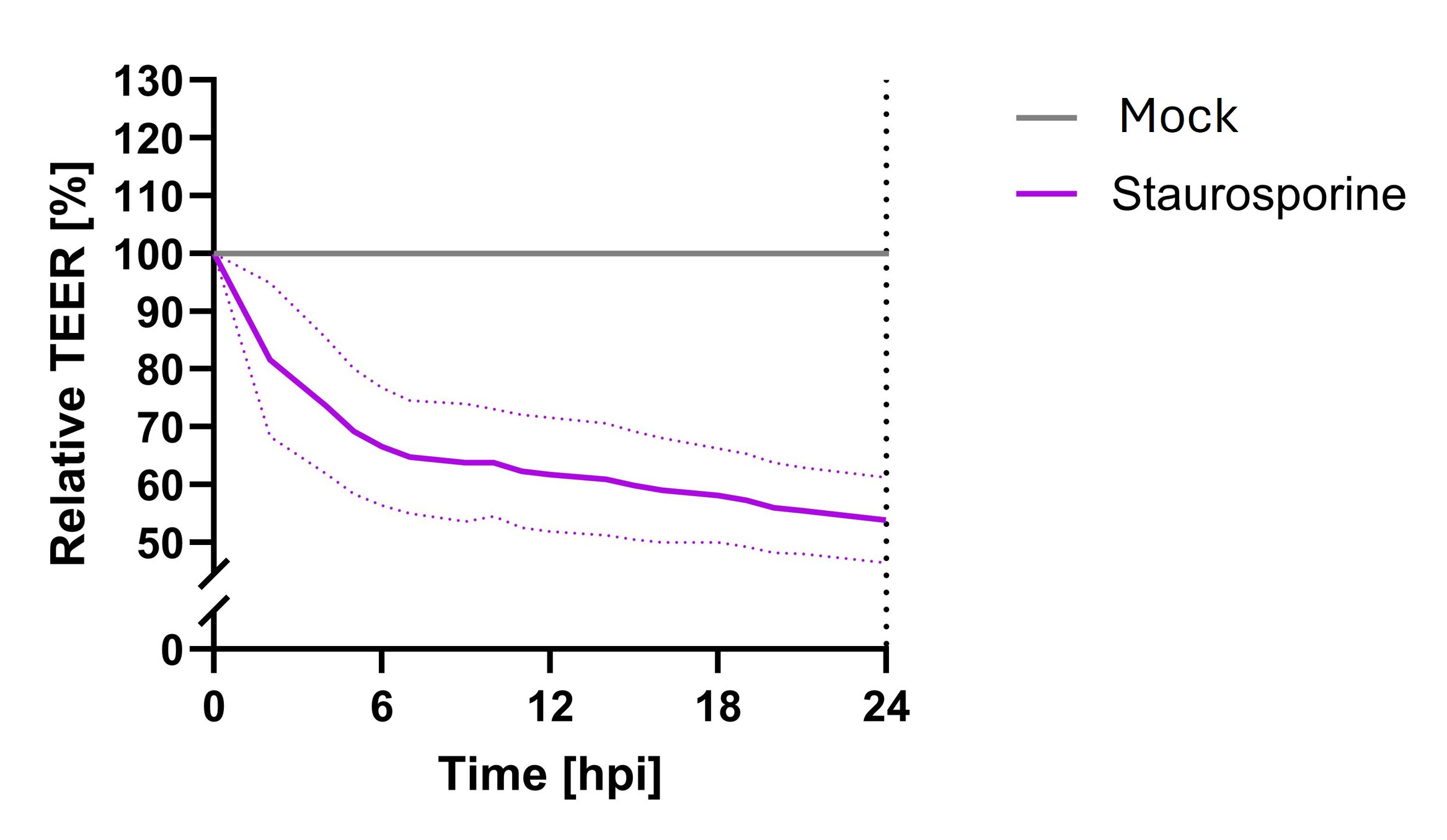

Supplement: Supplementary file 4 [file Image4.tif]

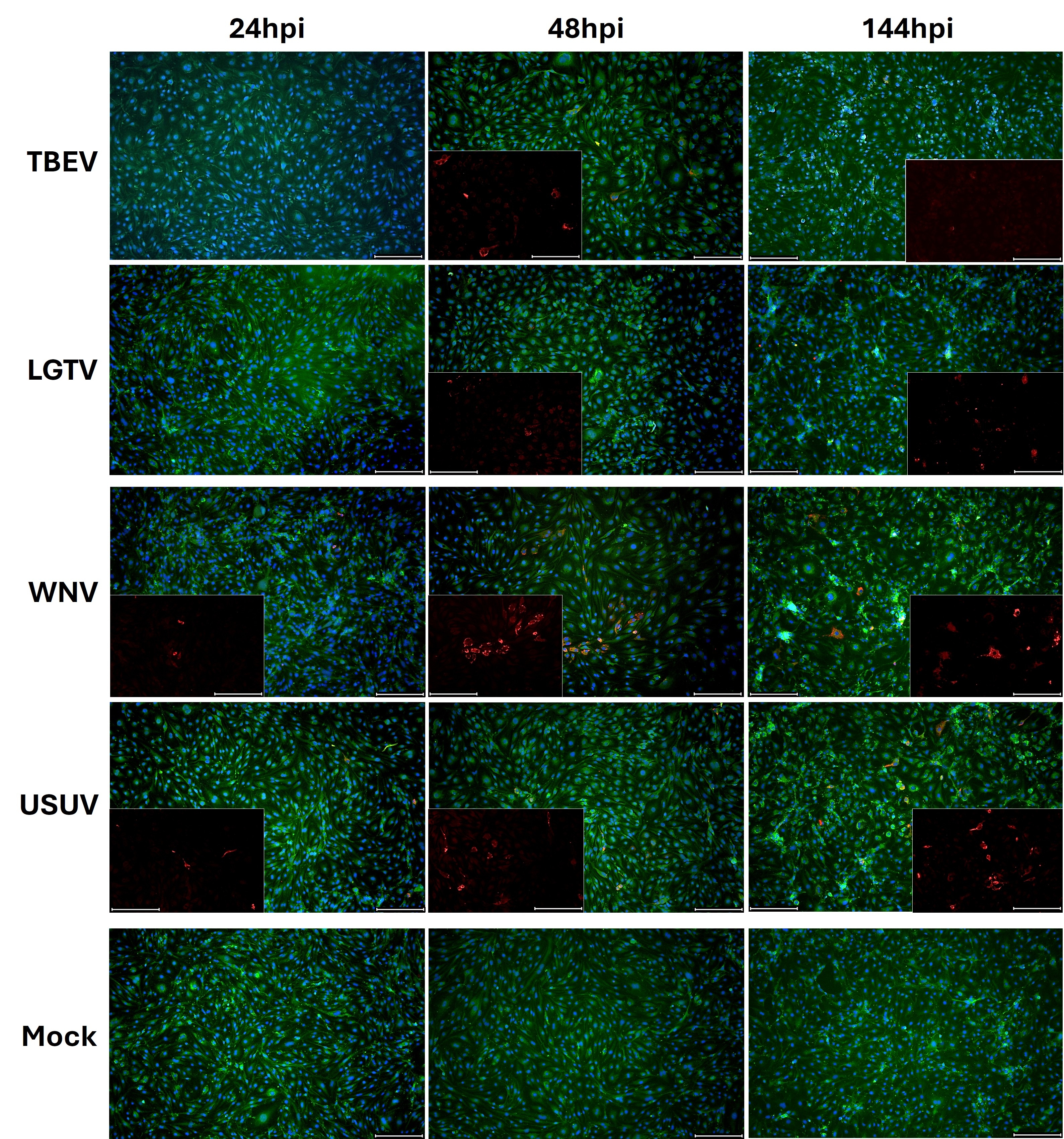

Supplement: Supplementary file 5 [file Image5.jpeg]
